# Molecular Epidemiology, Antimicrobial Resistance, and Virulence Profiles of *Staphylococcus aureus* from Fish, Aquatic Environments, and Fish Handlers in Southeast Nigeria

**DOI:** 10.3390/microorganisms13092059

**Published:** 2025-09-04

**Authors:** Uju Catherine Okafor, Onyinye Josephine Okorie-Kanu, Akwoba Joseph Ogugua, Chika Florence Ikeogu, Simeon Chibuko Okafor, Madubuike Umunna Anyanwu, Obichukwu Chisom Nwobi, Chidiebere Ohazuruike Anyaoha, Anthony Christian Mgbeahuruike, Lynda Onyinyechi Majesty-Alukagberie, Innocent Okwundu Nwankwo, Chukwunonso Francis Obi, Ejike Ekene Ugwuijem, Nkechi Harriet Ikenna-Ezeh, Ifeyinwa Riona Okosi, Onyemaechi Ugboh, George Okey Ezeifeka, Ekene Vivienne Ezenduka, Charles Odilichukwu R. Okpala, Edet Ekpenyong Udo

**Affiliations:** 1 Department of Veterinary Public Health and Preventive Medicine, University of Nigeria, Nsukka 402001, Nigeria; okaforujucatherine@gmail.com (U.C.O.); ogugua.akwoba@unn.edu.ng (A.J.O.); obichukwu.nwobi@unn.edu.ng (O.C.N.); chidi.anyaoha@unn.edu.ng (C.O.A.); lynda.majesty-alukagberie@unn.edu.ng (L.O.M.-A.); innocent.nwankwo@unn.edu.ng (I.O.N.); ejaikitomass@gmail.com (E.E.U.); ekene.ezenduka@unn.edu.ng (E.V.E.); 2 Department of Fisheries and Aquatic Management, Nnamdi Azikiwe University, Awka 420231, Nigeria; cf.ikeogu@unizik.edu.ng; 3 Department of Veterinary Pathology, University of Nigeria, Nsukka 402001, Nigeria; simeon.okafor@unn.edu.ng; 4 Department of Veterinary Microbiology and Immunology, University of Nigeria, Nsukka 402001, Nigeria; madubuike.anyanwu@unn.edu.ng (M.U.A.); anthony.mgbeahuruike@unn.edu.ng (A.C.M.); nkechi.ikenna-ezeh@unn.edu.ng (N.H.I.-E.); 5 Department of Veterinary Parasitology and Entomology, University of Nigeria, Nsukka 402001, Nigeria; chukwunonso.obi@unn.edu.ng; 6 Department of Diagnostic and Outstation Services, National Veterinary Research Institute, P.O. Box 01, Vom 930101, Nigeria; ify.okosi@nvri.gov.ng; 7 Department of Agricultural Economics and Extension, University of Delta, Agbor P.O. Box 2090, Nigeria; onyemaechi.ugboh@unidel.edu.ng; 8 Department of Veterinary Microbiology and Parasitology, College of Veterinary Medicine, Michael Okpara University of Agriculture, Umudike 440101, Nigeria; george.ezeifeka@mouau.edu.ng; 9 UGA Cooperative Extension, College of Agricultural and Environmental Sciences, University of Georgia, Athens, GA 30602, USA; 10 Department of Microbiology, Faculty of Medicine, Kuwait University, Kuwait City 12037, Kuwait; udo.ekpenyong@ku.edu.kw

**Keywords:** antimicrobial resistance, aquaculture, clonal complex, methicillin-resistant *Staphylococcus aureus*, molecular epidemiology, virulence genes

## Abstract

**Background:** *Staphylococcus aureus* is a major zoonotic and foodborne pathogen with substantial One Health implications, yet its prevalence, resistance, and virulence potential within the aquaculture sector in Nigeria remains poorly characterized. **Objectives**: To supplement existing information, this current study investigated the prevalence, clonal distribution, antimicrobial resistance, and virulence gene profiles of *S. aureus* isolates from fish, fish water, and occupationally exposed fish handlers in Anambra State, Southeast Nigeria. **Methods**: A total of 607 samples—comprising 465 surface swabs from raw and processed fish, 36 fish water samples, and 106 nasal swabs from fish handlers—were processed using selective culture, biochemical tests, antimicrobial susceptibility testing, DNA microarray analysis, *spa* typing, and SCC*mec* typing. **Results**: *S. aureus* was recovered from 16.5% (100/607) of the samples. Fourteen (14%) isolates were methicillin-resistant (MRSA), harboring *mecA* and SCC*mec* types IV and V, with a combined MRSA prevalence of 2.3%. Multidrug resistance was observed in 52.2% of isolates (mean Multiple Antimicrobial Resistance index: 0.23), with 19 resistance genes spanning nine antimicrobial classes—including heavy metal and biocide resistance. Twenty-eight *spa* types across 13 clonal complexes (CCs) were identified, with CC1, CC5, and CC8 predominating. The detection of shared *spa* types between fish and handlers indicates potential cross-contamination. Detected virulence genes included those for accessory gene regulators (*agrI*-*IV*), Pantone–Valentine leucocidin (*lukFS-PV*), toxic shock syndrome (*tsst-1*), hemolysins (*hla*, *hlb*, *hld*/*hlIII*, *hlgA*), biofilm formation (*icaA*, *icaD*), immune evasion (*chp*, *scn*, *sak*), enterotoxins (*sea*, *seb*, *sec*, *sed*, *egc*, and others), exfoliative toxins (*etA*, *etB*), epidermal cells differentiation (*edinA*, *edinB*), and capsular types (*cap5*, *cap8*). **Conclusions**: This study reveals that the aquaculture sector in Southeast Nigeria serves as a significant reservoir of genetically diverse, multidrug-resistant *S. aureus* strains with robust virulence profiles. These findings highlight the necessity of integrated One Health surveillance and targeted interventions addressing antimicrobial use and hygiene practices within aquatic food systems.

## 1. Introduction

*Staphylococcus aureus* is a highly adaptable bacterium that colonizes the skin and mucous membranes of humans and animals. It can cause a wide range of infections, including skin and soft tissue infections, septicemia, food poisoning, and bone and joint diseases. Its pathogenicity is driven by an array of virulence factors regulated by global systems such as the accessory gene regulator (agr) and staphylococcal accessory regulator (sar) [1]. These systems control the expression of structural and secreted virulence factors—including biofilms, hemolysins, Panton–Valentine leukocidin (PVL), exfoliative toxins, epidermal cell differentiation inhibitors (EDINs), and toxic shock syndrome toxin-1 (TSST-1)—that facilitate colonization, immune evasion, and host tissue damage [1,2]. Foodborne *S. aureus* is among major causes of staphylococcal food poisoning (SFP), characterized by abdominal cramps, vomiting, diarrhea, and fever. This condition results from ingestion of preformed, heat-stable enterotoxins that act as superantigens that disrupt the immune homeostasis by bypassing antigen presentation, triggering excessive cytokine production and impairing adaptive immune responses [3]. Toxigenic *S. aureus* strains may enter the food chain through contamination by infected or colonized food handlers, respiratory secretions, or unsanitary processing environments [4]. Although SFP is typically self-limiting, it can cause severe illnesses requiring hospitalization. However, its true incidence is often underestimated due to misdiagnosis, underreporting, or its sporadic occurrence [3,4]. In Nigeria, as in many other developing countries, inadequate food safety regulations, poor sanitation, and improper food storage practices contribute to elevated rates of *S*. *aureus* food contamination and bacterial proliferation [5,6]. SFP contributes to both direct and indirect economic losses, with disproportionately severe impacts in low- and middle-income countries [5]. While the economic burden of SFP appear underestimated at US$57,670 annually, the global economic impact of foodborne diseases projects to reach approximately US$95.2 billion per year, driven predominantly by healthcare expenditures and legal/reputational consequences as well as productivity losses [5,7].

Importantly, the clinical impact of *S. aureus* is compounded by its capacity to acquire antimicrobial resistance genes (ARGs), including those conferring resistance to last-line agents such as methicillin and vancomycin. For instance, those of methicillin resistance, encoded by the *mecA* or *mecC* genes within the staphylococcal cassette chromosome *mec* (SCC*mec*), renders the organism resistant to virtually all β-lactam antibiotics. SCC*mec* types I–XV can also harbor genes for resistance to other antimicrobials and heavy metals, further complicating treatment. Both methicillin-resistant (MRSA) and methicillin-susceptible *S. aureus* (MSSA) strains are frequently multidrug-resistant (MDR), representing a serious global health challenge. Between 2019 and 2021, *S. aureus* contributed to an estimated one million of the 127 million deaths associated with antimicrobial resistance globally [8,9,10]. In response, the World Health Organization (WHO) has classified *S. aureus* as a priority pathogen for surveillance and control within the One Health framework [11]. The organism’s ability to form biofilms exacerbates treatment difficulty, shielding it from antibiotics and host defenses [1,12]. Molecular tools such as DNA microarrays enable high-resolution genotyping of *S. aureus* via *spa* typing, multi-locus sequence typing (MLST), SCC*mec* classification, *agr* typing, and detection of virulence and resistance genes. These methods help characterize transmission dynamics across healthcare, community, and animal settings. Based on genotypic features, MRSA strains are categorized into healthcare-associated (HA-MRSA), community-associated (CA-MRSA), or livestock-associated (LA-MRSA) lineages. LA-MRSA, commonly of clonal complex (CC) 398, has been found in both animal products and community settings. SCC*mec* types I–III are primarily linked with HA-MRSA, while types IV and V are associated with CA-MRSA [13]. The *agr* system—a quorum-sensing regulator comprising RNAII (*agrB*, *agrD*, *agrC*, *agrA*) and RNAIII transcripts—controls biofilm formation and toxin expression. Four *agr* types (I–IV) are known, with distinct virulence patterns associated with each [14].

While studies from Egypt [15], South Africa [16], Asia [13,17,18], and Europe [19,20,21] have profiled *S. aureus* from fish and aquatic environments, the data specific to Nigeria appear limited. More so, most local (food) safety/epidemiological related studies in Nigeria have largely focused on human, animal, and animal-derived food samples [6], with minimal molecular characterization of isolates specific to fish and related environments [22]. Although fish are not natural hosts for staphylococci, contamination can occur via polluted water, human contact, or poor hygiene during handling and processing [4,23]. This situation is particularly applicable to Nigeria, where the unregulated antimicrobial use in aquaculture and widespread water pollution from industrial and agricultural sources [24,25], foster the selection and spread of resistant pathogens. In addition, fish remains a dietary staple in Nigeria, contributing to about 40% of the national protein intake, with per capita consumption estimated at 13.3 kg/year [26]. Traditional preservation methods such as smoking and oven-drying, often carried out under suboptimal hygienic conditions, increase the risk of *S. aureus* contamination and SFP. As the consumption of raw or undercooked fish [13] continues to emerge, this risk would further exacerbate. Occupational exposure in aquaculture settings combined with the use of fishpond effluents for irrigation [25] facilitates the environmental spread of *S. aureus* to humans, food crops, and wider ecosystem. Indeed, understanding the association between *S. aureus* antimicrobial resistance patterns, clonal lineages, and virulence gene profiles is essential for designing targeted public health interventions and informing empirical therapy. Therefore, to supplement existing information, this current study aimed to investigate the prevalence, molecular characteristics (including clonal structure, resistance determinants, and virulence profiles), and antimicrobial susceptibility patterns of *S. aureus* isolates from raw farmed and captured African catfish (*Clarias* spp.), fish water (fishpond, fish processing and river water), and occupationally exposed fish handlers in Anambra State, Southeast Nigeria.

## 2. Materials and Methods

### 2.1. Schematic Overview of the Experimental Program

**Figure 1:**
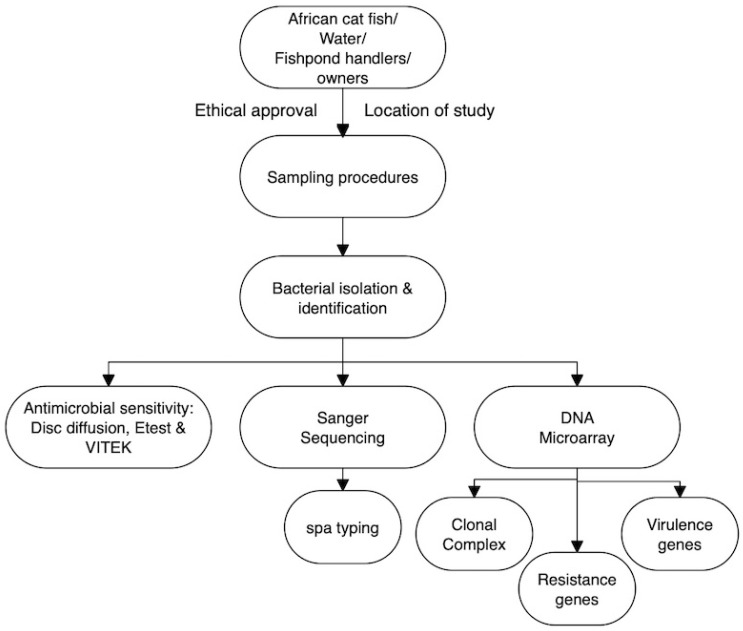
The schematic overview of the experimental program showing the major stages of this current work, from the targeted African catfish, water from the fishponds, and the handlers/owners (of those fishponds), through sampling procedures, to the bacterial isolation, identification, and subsequent analytical/characterization. Etest and VITEK = ETEST^®^ &VITEK brand associated with bioMérieux; *spa* typing = Staphylococcal Protein A (spa) Typing (Figure drawn using Flowchart Designer 5 Software Version 1.2.25, Apple Inc., California, USA).

The schematic representation of the experimental program (Figure 1) illustrates the principal stages of this study, beginning with the sampling of African catfish, pond water, and fish handlers/owners, and extending through bacterial isolation, identification, and subsequent analytical characterization. This research was specifically designed to investigate the prevalence, clonal distribution, antimicrobial resistance, and virulence gene profiles of *Staphylococcus aureus* isolates derived from fish, aquaculture water, and occupationally exposed handlers in Anambra State, Southeast Nigeria. The selected sampling sites were considered representative of the region, and all sampling procedures were conducted in accordance with ethical guidelines and good laboratory practices established by the Department of Veterinary Public Health and Preventive Medicine, University of Nigeria, Nsukka. Furthermore, all chemicals employed in this study were of analytical grade and sourced from certified suppliers.

### 2.2. Ethical Approval

This study received ethical clearance from the Institutional Animal Care and Use Committee of the Faculty of Veterinary Medicine, University of Nigeria (Protocol No. FVM-UNN-IACUC-2019–0143, approved 5 January 2019) and the Ethics Committee of Chukwuemeka Odimegwu Ojukwu University Teaching Hospital, Awka (Protocol No. COOUTH/CMAC/ETH.C/VOL.1/FN:04/243, approved 15 May 2019). The study was conducted in accordance with the principles outlined in the Declaration of Helsinki (2024) [27]. Prior to sample collection, both verbal and written informed consent were obtained from all fishpond owners and fish handlers who participated in the study.

### 2.3. Sampling

**Figure 2:**
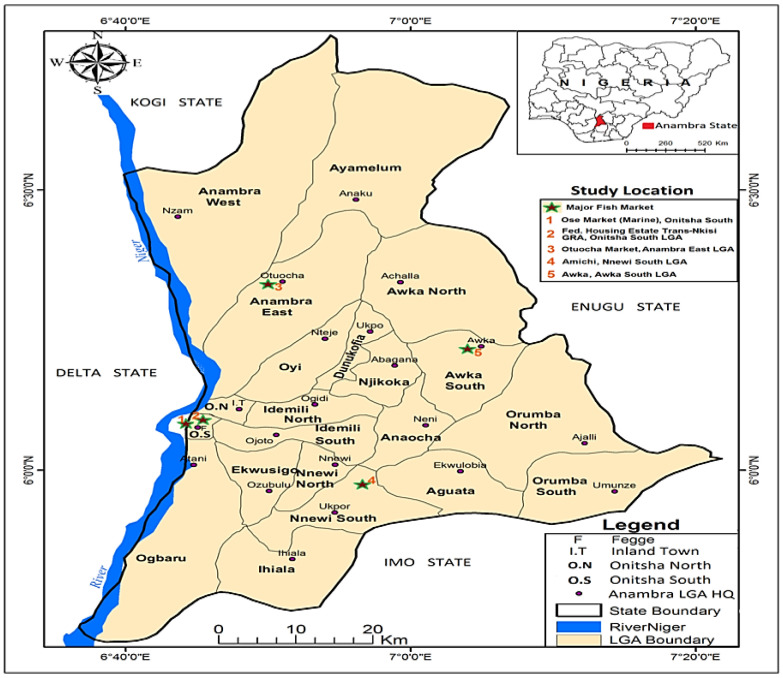
Map of Anambra State, Nigeria showing Local Government Areas and fishing communities (star 1–5) where fish, fish waters, and fish handlers were sampled.

A cross-sectional study was conducted between October 2019 and February 2020 across three Local Government Areas (LGAs), each representing one senatorial zone of Anambra State, Nigeria (Figure 1). The LGAs were selected based on the presence of fish farms, markets, and processing facilities. In each LGA, one major town—Awka, Nnewi, and Onitsha—was selected based on its notable involvement in African catfish (ACF; *Clarias* spp.) farming and oven-drying, and one fish farm was chosen from each town. Additionally, two fishing communities, Ose and Otuocha, where only smoking is used for fish preservation, were included (Figure 2). Weekly visits were made to these towns, during which a total of 465 ACF were randomly sampled. These included 246 freshly captured fish—64 farmed fish, 138 smoked captured fish, and 17 oven-dried farmed fish. Additionally, 36 water samples were collected, which included 24 from rivers and 12 from fishponds. A total of 106 occupationally exposed individuals involved in fish processing (70 using smoking and 36 using oven-drying were also randomly selected for sampling after obtaining written informed consent. About 5 cm^2^ area of the skin and freshly cut surfaces in raw fish and processed fish samples were swabbed using sterile swabs. Water samples were aseptically collected by immersing autoclaved 100 mL wide-mouth bottles with screw caps directly into the water (fish-holding [river and fishpond] and fish-processing waters) using gloved hands. Nasal swabs were obtained from each of the 76 fish handlers using sterile swabs. All fish and human samples represented approximately 5% and 50%, respectively, of the total individuals and fish available during the study period. Samples were transported on ice to the Microbiology Laboratory, Department of Veterinary Public Health and Preventive Medicine, University of Nigeria, and processed either immediately or within 24 h of collection for the isolation of *Staphylococcus aureus*. Importantly, all sampling procedures adhered to good laboratory practices prescribed by the Department of Veterinary Public Health and Preventive Medicine, University of Nigeria, Nsukka, Nigeria.

### 2.4. Bacterial Isolation and Identification

Swab samples were selectively enriched in 5 mL nutrient broth (Oxoid, Basingstoke, UK) supplemented with 6.5% NaCl and incubated aerobically at 35 ± 2 °C for 24 h. Following incubation, a loopful of the enriched culture was streaked onto Baird-Parker Agar (BPA) (Oxoid, Basingstoke, UK) containing egg yolk tellurite (EYT) and incubated in ambient air at 35 ± 2 °C for 48 h. Water samples (100 mL) were filtered through 0.45 µm membranes, which were placed directly on BPA and similarly incubated. Presumptive *S. aureus* colonies (black, shiny colonies with clear halos, with or without opaque zones) were sub-cultured on BPA with EYT for purification. Purified colonies were subjected to Gram staining and tested using catalase, slide and tube coagulase, and *S. aureus*-specific latex agglutination (Pastorex Staph-plus, Bio-Rad, Marnes-la-Coquette, France). Hemolysis was assessed on 5% sheep blood agar incubated aerobically at 35 ± 2 °C for 24 h. Isolates phenotypically identified as *S. aureus* were further confirmed and characterized using DNA microarray analysis capable of detecting 330 genetic determinants. For emphasis, the DNA microarray analysis was conducted at the Department of Microbiology, Faculty of Medicine, Kuwait University, Safat, Kuwait.

### 2.5. Antimicrobial Susceptibility Testing

Antimicrobial susceptibility testing of *S*. *aureus* isolates was conducted using the disk diffusion method, following the guidelines of the Clinical and Laboratory Standards Institute (CLSI M100-ED35:2025) [28]. This method was selected because *S. aureus* is not covered under the aquatic-specific CLSI VET03/VET04 standards [29,30]. A total of 16 antimicrobial agents representing 11 different classes were tested using antibiotic-impregnated disks (Oxoid, Hampshire, UK): β-lactams—penicillin (10 units) and cefoxitin (30 μg), aminoglycosides—gentamicin (10 μg), fluoroquinolones—ciprofloxacin (5 μg), macrolides—erythromycin (15 μg), lincosamides—clindamycin (2 μg), lipoglycopeptides—teicoplanin (30 μg), phenicols—chloramphenicol (30 μg), tetracyclines—tetracycline (10 μg), ansamycins—rifampicin (5 μg), folate pathway inhibitors—trimethoprim (5 μg), and monoxycarbolic acid—mupirocin (200 μg). In addition, amikacin (30 μg, aminoglycoside) and fusidic acid (10 μg; fusidanes steroidal antibiotic), and tigecycline (glycylcycline class) were, respectively, tested using the disk diffusion and minimum inhibition concentration (MIC) methods as per the European Committee on Antimicrobial Susceptibility Testing (EUCAST v.15, 2025) guidelines [31]. Isolates were adjusted to a 0.5 McFarland turbidity standard (approximately 1.5 × 10^8^ colony-forming unit (CFU)/mL) and uniformly spread onto Mueller–Hinton agar plates (Oxoid, Hampshire, UK). Antibiotic disks were applied within 15 min of inoculation, ensuring a minimum spacing of 24 mm (center to center). All plates were incubated aerobically at 35 ± 2 °C: minimum inhibitory concentrations (MICs) for cefoxitin, vancomycin (glycopeptides class), and teicoplanin were determined using ETEST^®^ strips (bioMérieux, Marcy l’Étoile, France) in accordance with the manufacturer’s instructions and the results interpreted as per the CLSI guidelines [28]. Inducible clindamycin resistance was assessed by the D-test [28]. Briefly, a 0.5 McFarland suspension of each isolate was spread onto Mueller–Hinton agar, and erythromycin (15 μg) and clindamycin (2 μg) disks were placed 15–26 mm apart within 15 min. Plates were incubated at 35 ± 2 °C for 18–24 h. The appearance of a D-shaped zone of inhibition adjacent to the erythromycin disk indicated inducible clindamycin resistance. Alternatively, hazy growth around the clindamycin disk, in the absence of a D-zone, was interpreted as resistance [28]. *Staphylococcus aureus* ATCC 25923 was used as a quality control strain for disk diffusion testing while *S*. *aureus* ATCC 29213 was used as control for MIC determination. The multiple antibiotic resistance index (MARI) was calculated for each isolate using the formula *a/b*, where *a* is the number of antibiotics to which the isolate was resistant, and *b* is the total number of antibiotics tested. Isolates resistant to three or more antimicrobial classes were classified as multidrug-resistant (MDR) [32].

### 2.6. Molecular Typing of Staphylococcus aureus Isolates

#### 2.6.1. Extraction of Genomic DNA for Amplification

*S*. *aureus* genomic DNA was obtained from a 24 h-old culture on 5% Columbia blood agar (Oxoid, Hampshire, UK). A prelysis step was conducted as previously described [33]. Thereafter, the DNA was extracted using the DNeasy Blood and Tissue Kit (Qiagen, Hilden, Germany) according to the manufacturer’s protocol

#### 2.6.2. Staphylococcal Protein A (spa) Typing and DNA Microarray Analysis

*spa* typing was performed by sequencing the hyper-variable region of the protein A gene (*spa*) following a previously described method [34,35]. The DNA microarray analysis was used to assess the presence of antimicrobial resistance genes and to assign the isolates to clonal complexes (CCs) using the *S*. *aureus* Genotyping Kit 2.0 system microarray-based assay (Abbott Rapid Diagnostics GmbH, Jena, Germany). The DNA microarray was performed as described by Monecke et al. [36,37].

### 2.7. Statistical Analysis

The frequencies of occurrence of *S*. *aureus*, *spa* types, clonal complexes, SCC*mec* types, resistance and virulence genes, and phenotypic resistance of the isolates to antimicrobial agents were entered into Microsoft Excel version 2010 (Microsoft Corporation, Redmond, USA) and subjected to descriptive statistics to determine their percentages. Associations between categorical variables were assessed using IBM SPSS Statistics version 31, applying two-tailed Fisher’s exact tests for independence in 2 × 2 contingency tables. Statistical significance was determined at a threshold of *p* < 0.05.

## 3. Results

### 3.1. Prevalence of Staphylococcus aureus

Out of 607 samples comprising fish (*n* = 465), fish waters (*n* = 36), and nasal swabs from fish handlers (*n* = 106), 100 non-duplicate *S. aureus* isolates were recovered, resulting in an overall prevalence of 16.5% (100/607) (Table 1). Prevalence rates by source were 15.5% in fish, 5.6% in water, and 24.5% in fish handlers. No statistically significant association (*p* > 0.05) was observed between isolation rates and sample type.

Of the 100 isolates, 14 (14%) were resistant to cefoxitin and were classified as methicillin-resistant *S. aureus* (MRSA), yielding an overall MRSA prevalence of 2.3% (14/607) (Table 1). The source-specific MRSA prevalence was 2.4% in fish and 2.8% in fish handlers; none was detected in fish water. The remaining 86 isolates were methicillin-susceptible *S. aureus* (MSSA), corresponding to a 24% prevalence.

### 3.2. spa Typing and Distribution of Clonal Complex (CC) of Staphylococcus aureus Isolates

The 100 *S. aureus* isolates were assigned to 28 distinct *spa* types, with *t948* (*n* = 16; 16%) and *t311* (*n* = 12; 12%) being the most prevalent, collectively accounting for 28% of all isolates (Table 2). Other notable *spa* types included *t355* (8%) and *t091* (7%). Six isolates (6%) were typed as t094 and t5911, while five (5%) were typed as *t002* and *t3040*. Four isolates (4%) belonged to *t189*, and three each to *t174*, *t315*, *t044*, and *t1814*. Two isolates (2%) each were identified as *t064*, *t1476*, *t6675*, and *t605*, while one isolate (1%) each belonged to *t084*, *t934*, *t1931*, *t6275*, *t1299*, *t4690*, *t2723*, *t2049*, *t1400*, *t5227*, and *t693*.

**Figure 3:**
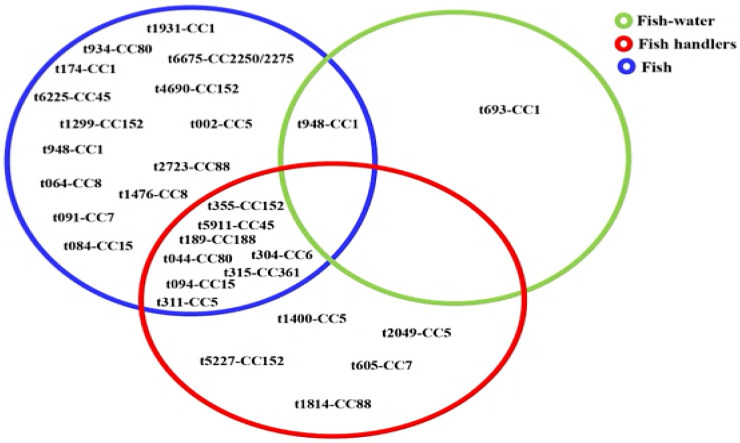
Distribution of 28 *spa-*clonal complexes of *Staphylococcus aureus* isolates from fish, fish waters, and fish handlers.

DNA microarray analysis grouped the isolates into 13 clonal complexes (CCs) (Table 2), with the majority belonging to CC1 (*n* = 21), CC5 (*n* = 19), and CC152 (*n* = 11). CC1 was detected in isolates from fish and fishpond water, whereas isolates representing other CCs (CC5, CC6, CC7, CC15, CC45, CC152, CC80, CC88, CC361, and CC188) were obtained from fish and fish handlers (Figure 3). Of the 72 isolates from fish, 23 *spa* types and 13 CCs were identified, while the 26 isolates from fish handlers comprised 13 *spa* types and 10 CCs. Only four *spa* types associated with three CCs were linked to MRSA isolates. These were *t091*-CC7 (*n* = 7), *t064*-CC8 (*n* = 2), and *t1476*-CC8 (*n* = 2) from fish, and *t1814*-CC88 from a fish handler. Of the 13 CCs, only CC88 included both MRSA and MSSA *spa* types. Notably, none of the *spa* types were shared between MRSA and MSSA isolates.

### 3.3. Antimicrobial Resistance Profile of Staphylococcus aureus Isolates

The antimicrobial susceptibility profiles of the 100 *S. aureus* isolates against 16 antimicrobial agents are summarized in Table 3. Resistance to penicillin was the most common (85%), followed by resistance to trimethoprim (69%), amikacin (51%), tetracycline (47%), clindamycin (34%), and erythromycin (32%). The lowest resistance rates were observed for gentamicin (23%), cefoxitin (14%), ciprofloxacin (11%), and chloramphenicol (6%). Overall, 92% of the isolates exhibited resistance to at least one antimicrobial agent. Statistically significant associations (*p* < 0.05) were observed between isolate type and resistance to cefoxitin, gentamicin, amikacin, erythromycin, clindamycin, chloramphenicol, tetracycline, trimethoprim, and ciprofloxacin (Table 3). Methicillin resistance (cefoxitin resistance) was detected in 14 isolates, which belonged to clonal complexes CC7 (*n* = 7), CC8 (*n* = 4), and CC88 (*n* = 3). None of the isolates exhibited resistance to tigecycline, rifampicin, mupirocin, fusidic acid, vancomycin, teicoplanin, and linezolid.

Among the 100 isolates, 8% were susceptible to all tested antibiotics. Resistance to one, two, three, four, six, seven, eight, and nine antimicrobial agents was observed in 12%, 32%, 14%, 3%, 3%, 15%, 7%, and 6% of isolates, respectively (Table 4) with the most common pattern being resistance to penicillin and trimethoprim (PEN-TRI, *n* = 16). Of the 92 resistant isolates, 49 (53.2%) demonstrated multiple drug resistance (defined as resistance to at least three antimicrobial classes). The predominant multiple drug resistance pattern among isolates from fish was PEN-GEN-AMK-ERY-CLI-TET-TRI (*n* = 13), while PEN-TRI (*n* = 6) predominated among those from fish handlers. Two distinct multiple drug resistance patterns were demonstrated by the isolates from fish water.

Overall, 52.2% (48/92) of the resistant isolates were classified as multidrug-resistant (MDR) (Table 4). The multidrug resistance prevalence among isolates from fish, fish handlers, and fish waters was 47.8% (33/69), 38.1% (8/21), and 100% (2/2), respectively (Table 4). The mean Multiple Antibiotic Resistance Index (MARI) across all isolates was 0.23 (range: 0.06–0.56). MRSA isolates exhibited the highest MARI values, ranging from 0.44 to 0.56. Notably, only isolates belonging to CC188 did not exhibit multidrug resistance (defined as resistance to at least one agent in three or more antibiotic classes).

### 3.4. Antimicrobial Resistance Genotypes of Staphylococcus aureus Isolates

A total of 19 antimicrobial resistance genes were identified among the 100 *S. aureus* isolates (Table 5). The *mecA* gene, associated with methicillin resistance, was detected in all 14 cefoxitin-resistant isolates. These *mecA*-positive isolates carried SCC*mec* types IV (*n* = 3) and V (*n* = 11). The efflux pump gene *sdrM* was the most frequently detected (98%), followed by β-lactamase-encoding genes *bla*Z, *bla*I, and *bla*R, present in 81% of the isolates. Resistance genes for chloramphenicol (*cat*, encoding chloramphenicol acetyltransferase) and trimethoprim (*dfrS1*, encoding dihydrofolate reductase) were found in 6% and 4% of the isolates, respectively. Tetracycline resistance genes, *tet*(K) (28%) and *tet*(M) (4%), occurred in 28% and 4% of the isolates, respectively. Aminoglycoside resistance genes were detected at varying frequencies: *aphA3* (11%), *aadD* (3%), and the bifunctional *aacA-aphD* (23%) encoding aminoglycoside phosphotransferase gene, aminoglycoside adenyltransferase, and aminoglycoside acetyltransferase–phosphotransferase enzymes, respectively. Macrolide resistance genes included the erythromycin ribosome methylase genes, *erm*(B) (18%), and *erm*(C) (13%), as well as the macrolide efflux gene, *msr*(A) (2%). In contrast, *Inu*(A), a lincosamide nucleotidyltransferase gene, was found in 8% of the isolates.

The phenotypic resistance profiles generally aligned with the genotypic findings. However, a macrolide–lincosamide-susceptible CC152-MSSA-t355 isolated from fish harbored both *erm*(C) and *InuA*, and a tetracycline-susceptible CC152-MSSA-t355 isolate carried *tet*(K). Genes conferring resistance to streptothricin, *sat* (encoding streptothricin acetyltransferase, 16%), and quaternary ammonium compounds (*qacC*, 5%), although not phenotypically assessed, were also detected. Notably, the prevalence of *blaZ*, *blaI*, *blaR*, *aacA-aphD*, *aphA3*, *erm*(C), *cat*, *sat*, *tet*(K), and *dfrS1* were significantly higher (*p* < 0.05) among MRSA compared to MSSA isolates (Table 5).

### 3.5. Prevalence of Virulence-Associated Genes in Staphylococcus aureus Isolates

#### 3.5.1. Regulatory Genes

**Figure 4:**
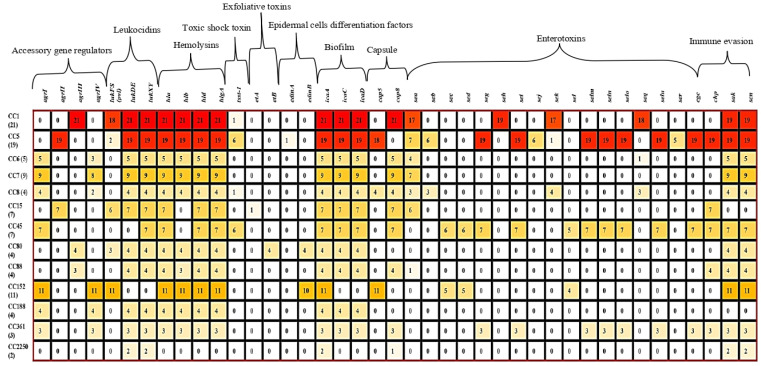
Tricolor scale depicting the association among *Staphylococcus aureus* virulence genes, *agr* types, and clonal complex. CC: clonal complex, *agr*: accessory gene regulator, *pvl*: Pantone–Valentine leucocidin, *luk*: Leucocidin, *hl*: Haemolysin, *et*: exfoliative toxin, *edin*: epidermal cells differentiation inhibitors, *ica*: intracellular capsular adhesion, *se*: *Staphylococcus* enterotoxin, *sak*: Staphylokinase, *chp*: chemotaxis inhibitory protein, *scn*: Staphylococcal complement inhibitor.

All 100 *S. aureus* isolates carried the regulatory genes *sarA*, *saeS*, and *vraS* (Table 6). Among the accessory gene regulator (*agr*) types, *agrI* was the most prevalent (43%), followed by *agrIV* (31%), *agrIII* (28%), and *agrII* (26%). The carriage of *agrI* was significantly higher (*p* = 0.007) in MRSA isolates (78.6%) compared to MSSA (37.2%) (Table 6). Similarly, *agrIV* was more frequently (*p* = 0.031) detected in MRSA (57.1%) than in MSSA (27.1%), while *agrII* was identified exclusively (*p* = 0.01816) in MSSA isolates (30.2%). All isolates belonging to CC45 and CC152 were positive for *agrI*, while CC1 and CC88 isolates were positive for *agrIII*. CC5, CC15, and CC80 isolates were positive for *agrII* (Figure 4). Isolates of CC6, CC7, CC8, CC152, CC188, and CC361 were positive for *agrI*. All CC2250 isolates were negative for all *agr* types (Figure 4).

#### 3.5.2. Virulence Genes

All isolates harbored at least two of the tested virulence-associated genes (Figure 4). The Pantone–Valentine leukocidin gene (*lukFS-PV*) was detected in 40% of the isolates, with a significantly higher (*p* = 0.00063) prevalence in MSSA (46.5%) compared to none in MRSA. Other leukocidin genes, *lukD/E* and *lukX/Y*, were detected in 84% and 88% of isolates, respectively (Table 6). The *lukFS-PV* gene was most common in CC152 (100%), CC1 (85.7%), CC15 (85.7%), and CC80 (75%), but was rare in CC5 (10.5%). All isolates carried *lukD/E* (except those of CC45 and CC152) and *lukXY* (absent in CC152) (Figure 4).

Nearly all isolates (98%) harbored hemolysin-encoding genes (*hla*, *hld/hlIII*, and *hlgA*), except for CC2250 (Figure 4). The *hlb* gene was the least frequent, present in 83% of all isolates, but was absent in CC15 and CC45 isolates (Table 6; Figure 4). Exfoliative toxin genes were rare. The *etA* was detected in 14.3% of CC15 isolates, and *etB* was detected in all CC8 isolates (Figure 4). These genes occurred exclusively in MSSA, accounting for 5% of the total isolates (Table 6). Moreover, genes encoding epidermal cell differentiation inhibitors were detected in 14% of isolates: *edinA* was found in one CC5 isolate (5.2%), while *edinB* was present in all CC80 and 90.9% of CC152 isolates. The TSST-1 gene, encoding toxic shock syndrome toxin, was identified in 14% of isolates, including 7.1% of MRSA and 15.1% of MSSA (Table 6). Its carriage was strongly associated with CC45 (85.7%), followed by CC5 (31.6%), CC8 (25%), and CC1 (4.8%) (Figure 4).

Classical enterotoxin genes were found at the following frequencies: *sea* (45%), *seb* (9%), *sec* (11%), and *sed* (6%). The *sea* gene was common in CC15 (85.7%), CC1 (81%), CC8 (75%), CC5 (36.8%), and CC88 (25%) (Table 3). *seb* was primarily associated with CC8 (75%), *sec* with CC45 (85.7%), and *sed* with both CC45 (85.7%) and CC152 (45.5%) (Figure 4). Other enterotoxin genes (*sek*, *seq*) were found in 22% of the isolates but showed no significant difference (*p* > 0.05) between MRSA and MSSA. Several non-classical enterotoxin genes occurred exclusively in MSSA, including *seg* (6%), *seh* (19%), *sei* (29%), *sej* (6%), *sel* (9%), *selm* (29%), *seln* (29%), *selo* (29%), *ser* (5%), and *selu* (29%) (Table 6). The prevalence of *sei*, *selm*, *seln*, *selo*, and *selu* was significantly higher (*p* = 0.00889) in MSSA compared to MRSA. These genes were mostly associated with CC5, CC45, and CC361, which were the only lineages carrying the enterotoxin gene cluster (*egc*) (Figure 4).

Immune evasion genes were detected as follows: *chp* (40%), *sak* (87%), and *scn* (94%) (Table 6). All MRSA isolates carried *sak* and *scn*, while *chp* was present in 21.4% of MRSA and 43.0% of MSSA isolates. CC188 isolates lacked all immune evasion genes; *chp* was absent in CC1, while *sak* and *scn* were absent in CC5 and CC45 isolates (Figure 4). Moreover, biofilm-associated genes (*icaA*, *icaC*, *icaD*) were prevalent across isolates. Both MRSA and MSSA showed 100% prevalence for *icaA*, while *icaC* and *icaD* were found in 87% and 98% of isolates, respectively (Figure 4). These genes were absent in isolates of CC152 and CC2250. Capsule-encoding genes *cap5* and *cap8* were detected in 33% and 65% of isolates, respectively (Table 6). *cap5* gene was found in over 90% of CC5 and CC8 isolates, while *cap8* was present in all isolates of CC1, CC6, CC7, CC8, CC15, CC45, CC88, CC152, and CC361 (Figure 4). No statistically significant association (*p* > 0.05) was found between *agr* type, source of isolation, and presence of virulent gene.

## 4. Discussion

This current study investigated the genetic diversity and antimicrobial resistance of *S*. *aureus* in aquaculture-associated environments (fish, fish handlers, and fish waters) within a local Nigerian fishery sector, through the One Health standpoint. The overall prevalence of *S. aureus* (16.5%) indicates a moderate yet concerning level of contamination. As *S. aureus* is not conventionally associated with aquatic environments, this finding implicates both fish and handlers as potential reservoirs and vectors, thereby underscoring their role in the human–animal–environment transmission cycle. The higher prevalence in fish (15.5%) and handlers (24.5%) compared to water (5.6%) likely reflects the post-harvest contamination, stemming from poor hygiene practices such as reuse of unwashed tools, inadequate hand hygiene, and lack of use of personal protective equipment. Although *Staphylococcus aureus* is not typically waterborne, its detection in fishpond water suggests anthropogenic contamination—potentially from farm runoff, human handling, or other environmental exposures. This raises environmental health concerns, as *S. aureus* can persist in aquatic environments and may enter the food chain through crops irrigated with fishpond effluents [38]. Compared to global reports from Iran [4], Egypt [15], Japan [39], Greece [21], and India [40], where *S. aureus* prevalence ranged from 0.4% to 27.83%, our findings fall within international trends. However, they are lower than the 52.1% and 57% reported in China [13] and Abuja Nigeria [41], respectively. The 24.5% carriage rate among (fish) handlers is particularly concerning, highlighting their critical role as conduits for pathogens’ introduction into aquatic ecosystems and potential dissemination along the food chain.

Moreover, the DNA genotyping in this current study revealed a substantial genetic diversity among the 100 *S. aureus* isolates, comprising 28 *spa* types distributed across 13 clonal complexes (CCs). This observation likely reflects the combined influence of multiple sources of contamination (humans, environment, and fish), selective pressures exerted by antimicrobial agents within the aquatic ecosystem that potentially stimulates the emergence of diverse lineages, and inadequate biosecurity measures that facilitate cross-contamination. These findings highlight the dynamic One Health interface inherent in aquaculture and underscore the potential of the Nigerian aquaculture sector to serve as a reservoir and dissemination pathway for diverse *S. aureus* lineages, some of which may carry zoonotic significance. The highest diversity was found in fish (23 *spa* types from 13 CCs) and handlers (13 *spa* types from 10 CCs). Most of these CCs, including CC1, CC8, CC15, CC5, and CC152, have been previously reported in food animals and human clinical isolates in Nigeria [42,43,44,45,46,47,48], and in both animal and human hosts globally [48,49,50,51,52,53,54], supporting their zoonotic potential. Notably, CC80 and CC88, which are well-documented in human and food animal infections in Nigeria [42,44], were also detected in this study, underscoring the interconnectedness of human, animal, and environmental reservoirs.

Further, there were several CCs associated with multiple *spa* types, for example, CC15 (t094, t084), CC8 (t064, t1476), and CC5 (t311, t002, t2049, t1400) were observed in both aquatic and human-related samples (Figure 3), which would be indicative of genetic heterogeneity and multiple transmission sources [35]. The detection of t311 (CC5)—a human-associated *spa* type linked to infections in Nigeria [55,56], which appeared dominant in healthcare settings in Ghana [57], and associated with bloodstream infections globally [58,59,60,61,62]—potentially in both fish and handlers would suggest human-to-fish transmission. Similarly, the most prevalent clone, t948 (CC1), identified in both fish and water, has been widely reported in clinical isolates across Africa [55,57,63] and Europe [62], indicating potential reverse zoonotic transmission. Other *spa* types such as t690, t355, t044, and t189 have also been reported in both human and animal isolates from Nigeria and various regions [41,44,46,50,51,60,64,65,66,67,68,69]. Eight MSSA *spa*-CC combinations, including t311-CC5, t355-CC152, and t044-CC80, appear as shared between fish and handlers, indicative of probable cross-contamination during handling or shared environmental sources [6]. Given that many of these lineages are primarily human-associated, their detection in aquaculture environments raises concern about the spillover of antimicrobial-resistant strains from humans to fish and the wider ecosystem.

Methicillin-resistant *S. aureus* (MRSA) was identified in both fish and handlers, albeit at a relatively low prevalence (2.3%). Nonetheless, the detection of *mecA*-positive strains, CC7-MRSA-V, CC8-MRSA-V, and CC88-MRSA-IV in distinct hosts suggests independent acquisition events and underscores the public health threat posed by aquaculture-related MRSA. These findings are consistent with previous reports of MRSA in food animals and humans in Nigeria [70,71]. The observed prevalence exceeds the 1% reported in fish/fish handlers in Borno State, Nigeria [22], but is lower than values from fish in Poland (29.5%) [72], Brazil (30%) [73], and India (50%) [40]. This study revealed epidemic and multidrug-resistant MRSA lineages such as ST7-MRSA-V-t091 (WA-MRSA-131), CC8-MRSA-V (t064, t1476), and CC88-MRSA-IV-t1814 (the WA-MRSA-2 lineage called ‘African clone’) in fish and fish handlers. These clones have been implicated in human infections across Nigeria and globally [44,45,47,74,75,76,77], underscoring their zoonotic and environmental adaptability. The dual detection of MRSA and MSSA variants of CC88 (t1814 and t2723, respectively) suggests genomic flexibility and selective pressure for SCC*mec* acquisition, likely driven by antimicrobial use. This aligns with earlier reports that CC88-MRSA is more prevalent than CC88-MSSA in Nigerian food animals [42]. The ST8-MRSA-V lineage, observed as multiple *spa* types (t064, t1814, t1417), further illustrates the widespread distribution of high-risk clones. This lineage has been reported in clinical samples from Nigeria [45,78], Sao Tome and Principe [79], hospital environments in Armenia [80], and poultry from southwestern Nigeria [71], highlighting its ability to adapt across hosts and environments.

Possibly, the situation of antimicrobial resistance appears widespread among the isolates of this current study. Specifically, the penicillin resistance (85%) was correlated with a high prevalence of β-lactamase-encoding genes *bla*Z, *bla*I, and *bla*R, possibly due to antibiotic misuse in both the aquaculture and human sectors in Nigeria [81,82]. Resistance to erythromycin (33%) and inducible clindamycin (32%) was linked to *erm*(B), *erm*(C), *Inu*(A), and *msr*(A), some of which appeared to be unexpressed (like the lincosamide-susceptible CC152-MSSA-t355 isolated from fish harbored both *ermC* and *InuA*), which would be suggestive of possible silent reservoirs. Tetracycline resistance (43%), predominantly mediated by *tet*(K), also likely emanated from its overuse in the human healthcare domain and aquaculture [80]. Notably, *tet(K)* was detected in a phenotypically susceptible isolate, suggesting the potential for cryptic resistance. Aminoglycoside resistance (14–47%) and low-level chloramphenicol resistance (6%) were linked to genes encoding known modifying enzymes (*aphA3*, *aadD*, and the bifunctional *aacA-aphD* encoding aminoglycoside phosphotransferase, aminoglycoside adenyltransferase and aminoglycoside acetyltransferase–phosphotransferase, and *ca*t encoding chloramphenicol acetyltransferase, respectively). In contrast, resistance to trimethoprim in most isolates were found to occur in the absence of dfrS1, implicating other determinants, such as *dfrG*, which appears not included in the microarray panel. Of particular concern is the detection of the fosfomycin resistance gene *fosB* in CC5, CC8, and CC15. Fosfomycin is a last-resort treatment for MDR *S. aureus*, and its resistance in aquaculture suggests either environmental contamination or antibiotic misuse.

The widespread presence of the *sdrM* efflux gene (98%) and the biocide resistance gene *qacC* (5%) further reflects environmental exposure to selective pressures from disinfectants and heavy metals used in fish farming, which raises important concerns about the co-selection of resistance. Preliminary phenotypic assays (disk diffusion screening) corroborate this, with high resistance to cadmium, mercury, and ethidium bromide. These findings have significant One Health ramifications, as resistant strains from aquaculture can spread into the broader environment and community through water, food, or direct human contact. Most resistance genes (except *tet*(M), *msr*(A), and *aadD*) were found in isolates carrying SCC*mec* types IV and V. The resistance genes are likely mediated by plasmids (such as pT181) or other mobile elements [43,83,84], facilitating their dissemination. The high rate of multidrug resistance (52.2%), with 90.2% of resistant isolates exhibiting resistance to two or more antimicrobial classes and mean MARI values exceeding 0.2 (0.23), underscores exposure to high-risk environments. MDR was particularly high in fish water (100%) and fish (47.8%) as well as in fish handlers (38.1%), reinforcing the notion that aquaculture practices in Nigeria may serve as hotbeds for antimicrobial resistance. Encouragingly, all isolates remained susceptible to critical ‘Reserve’ antibiotics like tigecycline, mupirocin, and vancomycin. Nonetheless, the detection of resistance to commonly used antimicrobials in all clonal complexes except CC88 is concerning. The dissemination of diverse *S. aureus* clones from aquaculture into the human population may compromise treatment efficacy and establish persistent reservoirs of resistance genes across ecosystems.

In this current study, the detection of universal regulators *sarA*, *saeS*, and *vraS* genes in all *Staphylococcus aureus* isolates underscores their conserved nature and critical role as global regulators across both MRSA and MSSA strains. These genes, encoding staphylococcal accessory regulator A, the sensor histidine kinase SaeS, and the sensor histidine kinase VraS, respectively, are key modulators of virulence gene expression, particularly those regulated by the agr quorum-sensing system [1]. Their ubiquitous presence across various strain types highlights their fundamental importance in regulating virulence. Given their role in biofilm formation, immune evasion, severe clinical manifestations such as sepsis, osteomyelitis, and necrotizing pneumonia [1,12], their presence represents a significant One Health concern.

Further, the detection of four distinct *agr* types with varying distributions between MRSA and MSSA isolates suggests that strain-specific regulatory mechanisms drive virulence. The *agr* locus, a central quorum-sensing regulator, facilitates biofilm dispersion, promotes intracellular survival, enhances dissemination through phagocytes, and induces the production of virulence factors such as proteases, hemolysins, and superantigens [2,85]. The significantly higher prevalence of *agrI* and *agrIV* among MRSA strains implies that carriage of multiple *agr* alleles may enhance the ability of these strains to acquire multidrug resistance determinants, consistent with the elevated resistance gene burden and high multiple antibiotic resistance (MAR) indices observed in MRSA. Interestingly, *agrII* was exclusively detected in MSSA isolates belonging to clonal complexes (CCs) 5, 15, and 80, suggesting lineage-specific regulatory adaptation. The detection of *agrIII* in both MRSA and MSSA (e.g., CC1, CC88, and CC7) indicates its broader distribution and potential involvement in the acquisition of resistance. The diversity of *agr* alleles among genetically distinct strains raises public health concerns, as *agr* regulation plays a central role in community-associated MRSA and MSSA infections [85]. Moreover, the potential for these lineages to share a common evolutionary origin can be reflected by the observation that strains within the same *agr* group often exhibit similar biological behavior and close genetic relatedness [86]. The lack of a significant association between *agr* types and virulence gene carriage, particularly in this current study, suggests that *agr* polymorphisms may influence phenotypic behavior such as resistance or persistence more than the mere presence of virulence genes, in line with previous findings [87]. The *agr*-deficiency observed in CC2250, along with reduced toxin production, suggests an atypical regulatory profile that may promote persistence rather than acute virulence. Dysfunction of the *agr* system has been associated with increased biofilm formation, impaired autolysis, and immune evasion, which together contribute to chronic infection and therapeutic failure [2]. The detection of *agr*-deficient strains in this study carries significant public health implications, as such strains have been linked to endocarditis, osteomyelitis, bacteremia, and increased mortality [85].

Of particular concern is the high detection rate (40%) of the Pantone–Valentine leucocidin gene (*lukFS-PV*) among MSSA isolates exclusively. This indicates that a substantial proportion of *S. aureus* strains circulating in the fishery sector may harbor this potent leukotoxin. PVL (Panton–Valentine leucocidin) is a bicomponent pore-forming toxin that contributes to leukocyte lysis and tissue necrosis, and is implicated in community-associated infections, including skin and soft tissue infections and necrotizing pneumonia [13]. The detection of PVL in MSSA from this sector represents a significant zoonotic risk to individuals who handle or consume contaminated fish. Comparative studies have reported varying prevalence of the PVL gene in MSSA from aquatic environments—100% in Egypt and 2% in India [15,40], and 18% detection rate in MRSA in South Africa [16], while a 50.4% detection rate was observed in aquatic products in China [13]. In Nigeria, the PVL gene has been previously reported in MRSA isolates from humans and food animals [42,88]. The significant association of PVL with MSSA in this study, especially among CC152, CC1, CC15, and CC80, reinforces the known link between PVL and community-associated lineages. These lineages have been implicated in human infections and are increasingly reported in community settings [1,85,89]. The widespread detection of other leucocidin genes, including *lukDE* and *lukXY*, further supports the strong cytolytic potential of the isolates. The near-universal presence of *lukD*, *lukE*, *lukX*, and *lukY* suggests their conservation and potential role in immune evasion and pathogenesis [90].

The high prevalence of *hla*, *hld*/*hlIII*, and *hlgA* (98%) underscores their conserved role in *S. aureus* virulence and immune evasion. Their absence only in CC2250 indicates universal distribution across CCs, suggesting evolutionary conservation. In contrast, the *hlb* gene, encoding β-hemolysin, was detected in only 83% of isolates and was absent in CC5, CC15, CC45, and CC80—human-adapted lineages previously associated with *hlb* disruption due to bacteriophage integration [90]. This lineage-specific absence likely reflects niche adaptation, as these CCs are often associated with colonization rather than invasive disease. The *hla* gene encodes α-hemolysin, which disrupts epithelial barriers, induces necrosis, and contributes to systemic infection, including sepsis and food poisoning outbreaks [13,85]. The *hld*/*hlIII* gene encodes δ-hemolysin, a component of the RNAIII regulatory system linked to the *agr* locus, known to lyse various host cells [90]. *hlb*, encoding β-hemolysin (a sphingomyelinase and non-pore-forming toxin), is implicated in inflammatory skin infections due to its activity as an epidermal growth factor receptor agonist [91]. *hlgA* encodes γ-hemolysin, a bicomponent leukocidin critical for survival in blood and implicated in serious infections such as bacteremia, septic arthritis, and toxic shock syndrome [90]. While workers like Rong et al. [13] reported *hla* and *hlb* detection rates of 89.4% and 6.7%, respectively, others like Sivaraman et al. [40] observed 0.78% for *hla* and 0% for *hlb*. Specific to Nigeria, Shittu et al. [40] detected all hemolysin genes in isolates from food animals.

Exfoliative toxin genes were detected in only 5% of MSSA isolates (CC15 and CC8), suggesting clonal restriction and vertical gene transmission [1]. Although uncommon in this setting, the presence of these serine proteases, which cause epidermal cell dissociation in staphylococcal scalded skin syndrome and bullous impetigo, is of clinical concern [92]. The detection of *edinA* and *edinB* in 14% of isolates—particularly the high prevalence of *edinB* in CC80 and CC152—indicates the emergence of virulent lineages within the aquatic food chain. EDINs disrupt epidermal cell development and have been implicated in pneumonia, bacteremia, and diabetic foot ulcers [93], warranting inclusion of these markers in AMR surveillance to monitor zoonotic risk. The 14% prevalence of TSST-1 among diverse isolates from fish and handlers suggests the potential for menstrual and non-menstrual toxic shock syndrome. TSST-1, a pyrogenic toxin superantigen, induces systemic inflammation via T-cell [93,94]. Its presence in fish and handlers raises public health concerns, especially for female handlers, even though foodborne toxic shock syndrome has not been confirmed. Global reports corroborate this: 3.9% prevalence in Iran [4], 12.5% in Egypt [15], 2.7% in China [13], and 6.25% in India [40]. In Nigeria, TSST-1 has been detected in *S. aureus* from ready-to-eat seafood [88].

Classical staphylococcal enterotoxins (SEA-SED), which are heat-stable superantigens associated with food poisoning [4], were detected in 4–45% of the isolates. This is particularly concerning given the increase in consumption of inadequately cooked smoked and roasted fish in Nigeria. *sea*, detected in 45% of isolates, was the most prevalent, consistent with global reports of SEA as the predominant enterotoxin in foodborne outbreaks [3]. Similar prevalences were recorded in fish sectors in Iran, Egypt, and South Africa [4,15,16] and in fishery products from China, India, and Spain [19,95,96]. The carriage of classical enterotoxin genes across multiple CCs suggests horizontal transmission, possibly via plasmids [3]. However, the exclusive presence of enterotoxin genes such as *seg*, *seh*, *sei*, *sej*, *sek*, *sel*, *selm*, *seln*, *selo*, *seq*, *ser*, and *selu* in MSSA isolates suggests a broader enterotoxin diversity within this group. The enterotoxin gene cluster (*egc*), containing several of these genes, was found only in CC5, CC45, and CC361, implying genomic island localization. Co-occurrence of *selm*, *seln*, selo, and *selu* in the same lineages suggest possible co-mobilization via pathogenicity islands [3].

Immune evasion cluster (IEC) genes—*scn* (94%), *chp* (60%), and *sak* (40%)—were moderately to highly prevalent, reflecting a likely human origin, as these genes are typically phage-encoded and human-specific [97]. Nonetheless, some lineages lacked these genes entirely: CC188 had none, *chp* was absent in CC1, and *sak*/*scn* were absent in CC5 and CC45. Their presence or absence reflects phage-mediated acquisition or loss and may indicate host-specific transmission dynamics [97]. Biofilm-associated genes were widely distributed, with *icaA* (100%), *icaC* (87%), and *icaD* (98%) being particularly prevalent in CC1, CC5, and CC361, indicating a strong biofilm-forming capacity across isolates. Biofilm formation enhances antimicrobial tolerance, persistence, and immune evasion, often contributing to treatment failure even in MSSA infections [98]. The universal presence of *icaA* highlights the conserved role of intercellular adhesion in early biofilm development [99]. The *ica*ADBC operon encodes enzymes critical for polysaccharide intercellular adhesin (PIA) synthesis and export [98,100]. Absence of *icaC* and *icaD* in CC152 and CC2250 suggests lineage-specific biofilm deficiencies, which seem consistent with previous findings that show CC152 as a poor biofilm producer as well as CC2250 being able to rely on alternative pathogenic mechanisms [65]. Capsule genes also show differential distribution with *cap8* (65%) being more prevalent than *cap5* (33%), consistent with global dominance of serotype 8 in both community-/hospital-associated strains [100]. While those of *cap5* appear limited to fewer lineages, those of *cap8* are broadly distributed across CC1, CC6, CC7, CC8, CC15, CC45, CC88, CC152, and CC361. Capsules enhance *S. aureus* virulence by suppressing phagocytosis, promoting intracellular survival, and supporting biofilm formation [100].

## 5. Conclusions

Raw and processed farmed or captured African catfish sold for human consumption in Anambra State, Southeast Nigeria, are potential reservoirs of diverse, virulent, multidrug-resistant (MDR), and methicillin-resistant *S. aureus* clones. These isolates form distinct molecular clusters and carry a broad arsenal of virulence and immune evasion genes. The local fish sector—often overlooked in zoonotic risk evaluations—may contribute to the environmental dissemination of human-adapted *S. aureus* strains via contaminated water, biofilm formation, or cross-contamination during handling and processing. This presents a significant One Health concern, particularly for individuals in direct contact with these fish, including farmers, processors, fishermen, animal health workers, vendors, and consumers. The findings underscore the urgent need for targeted surveillance, strict biosecurity measures, prudent antimicrobial use, and molecular monitoring in aquaculture systems to limit the emergence and spread of MDR *S. aureus*. Routine screening and coordinated One Health interventions are crucial for mitigating the zoonotic and public health risks associated with these resistant pathogens. 

Public health initiatives should emphasize antimicrobial stewardship, hygiene practices at both pre- and post-harvest stages, and responsible antibiotic use in both human and aquaculture settings. This study represents the first comprehensive genetic characterization of *S. aureus* from fish and the fishery sector in West Africa. A limitation of this current study would be the relatively small number of fish water samples and nasal swabs from fish handlers, which might likely restrict the ability to draw firm conclusions about the low prevalence of *S. aureus* and MRSA observed in these sources. Additionally, phenotypic assessment of virulence—particularly biofilm production—was not performed. This is critical, as the presence of the *ica* operon does not always correlate with expression, which may be influenced by environmental factors, genetic background, or the functionality of the *agr* system. Whole-genome sequencing (WGS) would have provided deeper insight into the genetic context of resistance and virulence genes, elucidating the mechanisms of acquisition, regulation, and transmission.

## Figures and Tables

**Table 1:** Prevalence of *Staphylococcus aureus* in fish, fish waters, and fish handlers.

Sample	Number	Number (%, 95% CI) of Isolates		
		*S*. *aureus*	MRSA	MSSA
Fish	465	72 (15.5, 12.2–18.8)	11 (2.4, 1.0–3.8)	61 (13.1, 10.0–16.2)
Fish waters	36	2 (5.6, 0.0–13.1)	0 (0.0, 0.0–0.0)	2 (5.6, 0.0–13.1)
Fish handlers	106	26 (24.5, 16.3–32.7)	3 (2.8, 0.0–6.0)	23 (21.7, 14.0–29.4)
Total	607	100 (16.5, 13.6–19.4)	14 (2.3, 1.1–3.5)	86 (14.2, 11.5–16.9)

CI: confidence interval, MRSA: methicillin-resistant *S*. *aureus*; MSSA: methicillin-susceptible *S*. *aureus*.

**Table 2:** Distribution of *spa* types and clonal complexes of *S. aureus* from fish, fish waters, and fish handlers.

Clonal Complex (Number)	*Spa* Types	Number (%) of Isolates			Total % Frequency (N = 100)
		Fish (N = 72)	Fish Handlers (N = 26)	Fish Water (N = 2)	
CC1 (21)	t948	15 (20.8)	0 (0)	1 (50)	16
	t174	3 (4.2)	0 (0)	0 (0)	3
	t1931	1 (1.4)	0 (0)	0 (0)	1
	t693	0 (0)	0 (0)	1 (50)	1
CC5 (19)	t311	8 (11.1)	4 (15.4)	0 (0)	12
	t002	5 (6.9)	0 (0)	0 (0)	5
	t2049	0 (0)	1 (3.8)	0 (0)	1
	t1400	0 (0)	1 (3.8)	0 (0)	1
CC6 (5)	t304	2 (2.8)	3 (11.5)	0 (0)	5
CC7 (9)	t091	7 (9.7) *	0 (0)	0 (0)	7
	t605	0 (0)	2 (7.7)	0 (0)	2
CC8 (4)	t064	2 (2.8) *	0 (0)	0 (0)	2
	t1476	2 (2.8) *	0 (0)	0 (0)	2
CC15 (7)	t084	1 (1.4)	0 (0)	0 (0)	1
	t094	3 (4.2)	3 (11.5)	0 (0)	6
CC45 (7)	t6225	1 (1.4)	0 (0)	0 (0)	1
	t5911	2 (2.8)	4 (15.4)	0 (0)	6
CC80 (4)	t044	2 (2.8)	1 (3.8)	0 (0)	3
	t934	1 (1.4)	0 (0)	0 (0)	1
CC88 (4)	t2723	1 (1.4)	0 (0)	0 (0)	1
	t1814	0 (0)	3 (11.5) *	0 (0)	3
CC152 (11)	t355	7 (9.7)	1 (3.8)	0 (0)	8
	t1299	1 (1.4)	0 (0)	0 (0)	1
	t4690	1 (1.4)	0 (0)	0 (0)	1
	t5227	0 (0)	1 (3.8)	0 (0)	1
CC188 (4)	t189	3 (4.2)	1 (3.8)	0 (0)	4
CC361 (3)	t315	2 (2.8)	1 (3.8)	0 (0)	3
CC2250/2277 (2)	t6675	2 (2.8)	0 (0)	0 (0)	2

*: Clonal complex and *spa* types of methicillin-resistant *Staphylococcus aureus*.

**Table 3:** Antimicrobial susceptibility profile of *Staphylococcus aureus* from fish, fish waters, and fish handlers.

Antimicrobial Class	Antimicrobial Agent	Number (%) of Resistant Isolates		% Frequency (N = 100)	*p* Value
		MRSA (N = 14)	MSSA (N = 86)		
β-lactams	Cefoxitin	14 (100)	0 (0.0)	14	0.000 *
	Penicillin	14 (100)	71 (82.6)	85	0.066
Aminoglycosides	Gentamicin	8 (57.1)	15 (17.4)	23	0.003 *
	Amikacin	13 (93)	38 (44.2)	51	0.000 *
Macrolides	Erythromycin	14 (100)	20 (23.3)	34	0.000 *
Lincosamides	Clindamycin	14 (100)	18 (20.9)	32	0.000 *
Phenicols	Chloramphenicol	5 (35.7)	1 (1.2)	6	0.000 *
Tetracyclines	Tetracycline	14 (100)	33 (38.4)	47	0.000 *
	Tigecycline	0 (0)	0 (0)	0	1.000 *
Folate pathway antagonists	Trimethoprim	14 (100)	55 (63.9)	69	0.004 *
Ansamycins	Rifampicin	0 (0)	0 (0)	0	1.000
Fluoroquinolones	Ciprofloxacin	8 (57.1)	3 (3.5)	11	0.000 *
Fusidanes steroidals	Fusidic acid	0 (0)	0 (0)	0	1.000
Monoxycarbolic acids	Mupirocin	0 (0)	0 (0)	0	1.000
Glycopeptides	Teicoplanin	0 (0)	0 (0)	0	1.000
	Vancomycin	0 (0)	0 (0)	0	1.000

MRSA: methicillin-resistant *Staphylococcus aureus*, MSSA: methicillin-susceptible *Staphylococcus aureus*, MSSA: methicillin-susceptible *Staphylococcus aureus*, *: significant difference across a row at *p* < 0.005.

**Table 4:** Antimicrobial resistance patterns and antimicrobial resistance indices of *Staphylococcus aureus* from fish, fish handlers, and fish waters.

SN	Antimicrobial Resistance Pattern	No. of Antimicrobials (MARIs)	Fish (N = 69)	Handlers (N = 21)	Water (N = 2)	Total (N = 92)	No. of Antimicrobial Classes	% Frequency of MDR Strains
1	PEN	1 (0.06)	7	1	0	8	1	52.2
2	TRI		1	0	0	1		
3	AMK		2	1	0	3		
4	PEN-TRI	2 (0.13)	10	6	0	16	2	
5	PEN-TET		3	3	0	6		
6	AMK-TRI		1	0	0	1		
7	AMK-CIP		1	0	0	1		
8	TET-TRI		3	0	0	3		
9	PEN-AMK		2	2	0	4		
10	PEN-CIP		1	0	0	1		
11	PEN-TET-TRI	3 (0.19)	4	0	0	4	3	
12	PEN-ERY-TRI		1	0	0	1		
13	PEN-CHL-TRI		1	0	0	1		
14	PEN-AMK-TRI		5	0	0	5		
15	PEN-TRI-CIP		1	0	0	1		
16	AMK-TET-TRI		0	1	0	1		
17	PEN-AMK-TET		0	1	0	1		
18	PEN-AMK-TET-TRI	4 (0.25)	0	0	1	1	4	
19	PEN-AMK-ERY-TRI		0	1	0	1		
20	AMK-ERY-CLI-TRI		0	1	0	1		
21	PEN-GEN-ERY-CLI-TET-TRI	6 (0.38)	1	0	0	1	6	
22	PEN-AMK-ERY-CLI-TET-TRI		1	1	0	2		
23	FOX-PEN-ERY-CLI-TET-TRI-CIP	7 (0.44)	1	0	0	1		
24	PEN-GEN-AMK-ERY-CLI-TET-TRI		13	0	1	14		
25	FOX-PEN-GEN-AMK-ERY-CLI-TET-TRI	8 (0.50)	1	0	0	1		
26	FOX-PEN-GEN-AMK-ERY-CLI-TRI-CIP		1	0	0	1		
27	FOX-PEN-AMK-ERY-CLI-CHL-TET-TRI		2	3	0	5	7	
28	FOX-PEN-GEN-AMK-ERY-CLI-TET-TRI-CIP	9 (0.56)	6	0	0	6		

MARIs: multiple antimicrobial resistance indices, N: total number, PEN: Penicillin, TRI: Trimethoprim, AMK: Amikacin, TET: Tetracycline, CIP: Ciprofloxacin, ERY: Erythromycin, CHL: Chloramphenicol, ERY: Erythromycin, CLI: Clindamycin, GEN: Gentamicin, FOX: Cefoxitin.

**Table 5:** Frequency of antimicrobial resistance genes in *Staphylococcus aureus* isolates from fish, fish waters, and fish handlers.

Antimicrobial Resistance Function	Resistance Gene	Number (%) of Positive Isolates		Total % Frequency (N = 100)	*p* Value
		MSSA (N = 86)	MRSA (N = 14)		
β-lactam determinants	*mec*A	0 (0)	14 (100)	14	0.000 *
	*bla*Z	67 (77.9)	14 (100)	81	0.041 *
	*bla*I	67 (77.9)	14 (100)	81	0.041 *
	*bla*R	67 (77.9)	14 (100)	81	0.041 *
Aminoglycoside determinants	*aacA-aphD*	14 (16.3)	9 (64.3)	23	0.000 *
	*aphA3*	5 (5.8)	6 (42.6)	11	0.001 *
	*aadD*	3 (3.5)	0 (0)	3	0.633
Macrolide–lincosamide determinants	*erm*(B)	17 (19.8)	1 (7.1)	18	0.455
	*erm*(C)	3 (3.5)	10 (71.4)	13	0.000 *
	*Inu*(A)	5 (5.8)	3 (21.4)	8	0.081
	*msr*(A)	2 (2.3)	0 (0)	2	1.000
Phenicol determinants	*cat*	1 (1.2)	5 (35.7)	6	0.000 *
Streptothricin determinants	*sat*	5 (5.8)	11 (78.6)	16	0.000 *
Fosfomycin determinants	*fosB*	34 (39.5)	4 (28.5)	38	0.558
Tetracycline determinants	*tet*(K)	14 (16.3)	14 (100)	28	0.000 *
	*tet*(M)	4 (4.7)	0 (0)	4	1.000
Trimethoprim determinants	*dfrS1*	0 (0)	4 (28.5)	4	0.000 *
Quaternary ammonium compound determinants	*qacC*	4 (4.7)	1 (7.1)	5	0.541
Efflux pump determinants	*sdrM*	84 (97.7)	14 (100)	98	1.000

MSSA: methicillin-susceptible *Staphylococcus aureus*, MRSA: methicillin-resistant *Staphylococcus aureus*, N: total number, *: implies significant difference across a row at *p* < 0.05.

**Table 6:** Prevalence of regulatory and virulence-associated genes in *Staphylococcus aureus* isolates from fish, fish waters, and fish handlers.

Function	Gene	Number (%) of Isolates Positive		Total % Frequency (N = 100)	*p* Value
		MRSA (N = 14)	MSSA (N = 86)		
Regulators	*agrI*	11 (78.6)	32 (37.2)	43	0.007 *
	*agrII*	0 (0)	26 (30.2)	26	0.018 *
	*agrIII*	3 (21.4)	25 (29.1)	28	0.752
	*agrIV*	8 (57.1)	23 (26.7)	31	0.031 *
	*sarA*	14 (100)	86 (100)	100	1.000
	*saeS*	14 (100)	86 (100)	100	1.000
	*vraS*	14 (100)	86 (100)	100	1.000
Leucocidins	*lukF*/*lukS* (*pvl*)	0 (0)	40 (46.5)	40	0.001 *
	*lukD*/*lukE*	14 (100)	70 (81.4)	84	0.117
	*lukX*/*lukY*	14 (100)	74 (86.0)	88	0.208
Haemolysins	*hla*	14 (100)	84 (97.7)	98	1.000
	*hlb*	14 (100)	69 (80.2)	83	0.119
	*hld/hlIII*	14 (100)	84 (97.7)	98	1.000
	*hlgA*	14 (100)	84 (97.7)	98	1.000
Toxic shock toxins	*tsst-1*	1 (7.1)	13 (15.1)	14	0.685
Exfoliative toxins	*etA*	0 (0)	1 (1.2)	1	1.000
	*etD*	0 (0)	4 (4.7)	4	1.000
Epidermal cell differentiation inhibitors	*edinA*	0 (0)	1 (1.2)	1	1.000
	*edinB*	0 (0)	14 (16.3)	14	0.208
Enterotoxins	*sea*	9 (64.3)	36 (41.9)	45	0.151
	*seb*	3 (21.4)	6 (7.0)	9	0.111
	*sec*	0 (0)	11 (12.8)	11	0.352
	*sed*	0 (0)	6 (7.0)	6	0.591
	*sek*	2 (14.3)	20 (23.3)	22	0.729
	*seq*	3 (21.4)	19 (22.1)	22	0.729
	*seh*	0 (0)	19 (22.1)	19	0.066
	*sej*	0 (0)	29 (33.7)	29	0.009 *
	*sej*	0 (0)	6 (7)	6	0.591
	*seg*	0 (0)	6 (7)	6	0.591
	*sel*	0 (0)	9 (10.5)	9	0.352
	*ser*	0 (0)	5 (5.8)	5	1.000
	*seln*	0 (0)	29 (33.7)	29	0.009
	*selm*	0 (0)	29 (33.7)	29	0.009 *
	*selo*	0 (0)	29 (33.7)	29	0.009 *
	*selu*	0 (0)	29 (33.7)	29	0.009 *
Hlb-converting phages/Immune evasion	*chp*	3 (21.4)	37 (43.0)	40	0.152
	*sak*	14 (100)	73 (84.9)	87	0.205
	*scn*	14 (100)	80 (93)	94	0.591
Capsule	*cap5*	4 (28.6)	29 (33.7)	33	1.000
	*cap8*	9 (64.3)	56 (65.1)	65	1.000
Biofilm	*icaA*	14 (100)	86 (100)	100	1.000
	*icaC*	14 (100)	73 (84.9)	87	0.205
	*icaD*	14 (100)	74 (86)	98	0.208

*agr*: accessory gene regulator, *sarA*: staphylococcal accessory regulator A, *saeS*: sensor histidine kinase SaeS, *vraS*: sensor histidine kinase VraS, *hld*: delta-hemolysin, lukF/lukS: Pantone–Valentine (lukF/S) leukocidin, *lukD/lukE*: LukDE toxin, *lukX/lukY*: LukXY toxin, *hl*: haemolysin alpha, beta, delta, *hlgA*: gamma haemolysin, *tsst*: toxic shock toxin, *et*: exfoliative toxin, *edin*:= epidermal differentiation inhibitor, *se*: staphylococcal enterotoxin, *chp*: chemotaxis inhibitory protein, *sak*: staphylokinase, *scn*: *: implies significant difference across a row at *p* < 0.05.

## Data Availability

The original contributions presented in this study are included in the article. Further inquiries can be directed to the corresponding author(s).
